# *Lactobacillus paracasei* KBL382 administration attenuates atopic dermatitis by modulating immune response and gut microbiota

**DOI:** 10.1080/19490976.2020.1819156

**Published:** 2020-10-04

**Authors:** Woon-Ki Kim, You Jin Jang, Dae Hee Han, Kyungchan Jeon, Cheonghoon Lee, Hyuk Seung Han, GwangPyo Ko

**Affiliations:** a Graduate School of Public Health, Seoul National University, Seoul, Republic of Korea; b Institute of Health and Environment, Seoul National University, Seoul, Republic of Korea; c N-Bio, Seoul National University, Seoul, Republic of Korea; d KoBioLabs, Inc., Seoul, Republic of Korea; e Center for Human and Environmental Microbiome, Seoul National University, Seoul, Republic of Korea

**Keywords:** *Lactobacillus paracasei*, atopic dermatitis, immunomodulation, microbiome, metabolome

## Abstract

Administration of probiotics has been linked to immune regulation and changes in gut microbiota composition, with effects on atopic dermatitis (AD). In this study, we investigated amelioration of the symptoms of AD using *Lactobacillus paracasei* KBL382 isolated from the feces of healthy Koreans. Mice with *Dermatophagoides farinae* extract (DFE)-induced AD were fed 1 × 10^9^ CFU d^−1^ of *L. paracasei* KBL382 for 4 weeks. Oral administration of *L. paracasei* KBL382 significantly reduced AD-associated skin lesions, epidermal thickening, serum levels of immunoglobulin E, and immune cell infiltration. *L. paracasei* KBL382-treated mice showed decreased production of T helper (Th)1-, Th2-, and Th17-type cytokines, including thymic stromal lymphopoietin, thymus, and activation-regulated chemokine, and macrophage-derived chemokine, and increased production of the anti-inflammatory cytokine IL-10 and transforming growth factor-β in skin tissue. Intake of *L. paracasei* KBL382 also increased the proportion of CD4+ CD25+ Foxp3+ regulatory T cells in mesenteric lymph nodes. In addition, administration of *L. paracasei* KBL382 dramatically changed the composition of gut microbiota in AD mice. Administration of KBL382 significantly ameliorates AD-like symptoms by regulating the immune response and altering the composition of gut microbiota.

## Introduction

The prevalence of atopic dermatitis (AD) is increasing worldwide.^1^ AD can be caused by numerous etiological factors,^2,3^ and typically characterized by the T helper (Th)2-dominated immune response.^4^ For example, IL-4, a key cytokine involved in atopic inflammation, is secreted from mast cells and drives the differentiation of T helper cells into Th2 cells. Further, it produces Th2-related cytokines and induces immunoglobulin (Ig)E isotype.^4,5^ Infiltration of inflammatory cells such as eosinophils and lymphocytes into skin lesions is regulated by Th2 chemokines^6,7^ and induced by IL-4, IL-5, and IL-13, potent eosinophil cytokines released from activated mast cells.^4,8,9^ In addition, Th2-derived IL-31 and thymic stromal lymphopoietin (TSLP) produced by keratinocytes can promote the sensation of itching, leading to scratching that further exacerbates skin eruption.^2,10,11^

In addition to Th2 cells, Th1 and Th17 cells are also associated with AD progression. The acute phase of AD occurs at the onset of Th1-cell activation.^12^ Also, Th17 cells play a role in the development of AD, acting as an initial cytokine source for the development of skin lesions.^13^ In AD patients, transepidermal water loss (TEWL) increases due to defects of the intercellular lipid bilayers in the stratum corneum.^14^

TSLP-stimulated dendritic cells (DCs) induce differentiation of naive T cells into Th2 cells and Th17 cells, leading to allergic inflammation of the skin.^15^ In addition, thymus and activation-regulated chemokine (TARC) and macrophage-derived chemokine (MDC), which are induced by keratinocytes, trigger Th2 development.^16,17^

Gut microbiota and their repertoire of biochemical reactions contribute to many aspects of host health, including metabolism, immunity, development, and behavior. Microbial dysbiosis, which is an imbalance of the microbial community, can contribute to the development of numerous diseases.^18^ Dysbiosis of gut microbiota is a driver of the development of autoimmune diseases such as AD.

Probiotics, also referred to as live biotherapeutic products (LBPs), are live microorganisms that can confer beneficial health effects on the host when administered in adequate amounts.^19^ Probiotics are a potential agent for preventing the symptoms of AD.^20^ Lactic acid bacteria are the most commonly used probiotics, with emerging applications for the treatment of various diseases, including AD.^21–23^ Previous studies have shown that oral administration of probiotics prevents the development of AD by inhibiting skin inflammation^4,24,25^ or through the generation of CD4+ Foxp3+ regulatory T (Treg) cells,^26^ which migrate to the skin from lymph nodes.^27^ Administration of certain *Lactobacillus* strains can prevent the development of skin legions in an AD model through induction of inhibitory cytokines such as IL-10.^24^ In addition, some *Lactobacillus* strains reverse gut dysbiosis-related diseases by regulating intestinal homeostasis.^28,29^

We investigated the effects of *Lactobacillus* strains on AD-like symptoms in terms of immunomodulation, the composition of cecum microbiota, and their metabolites.

## Results

### *KBL382 treatment alleviates* Dermatophagoides farinae *extract (DFE)-induced AD-like symptoms*

**Figure 1.**
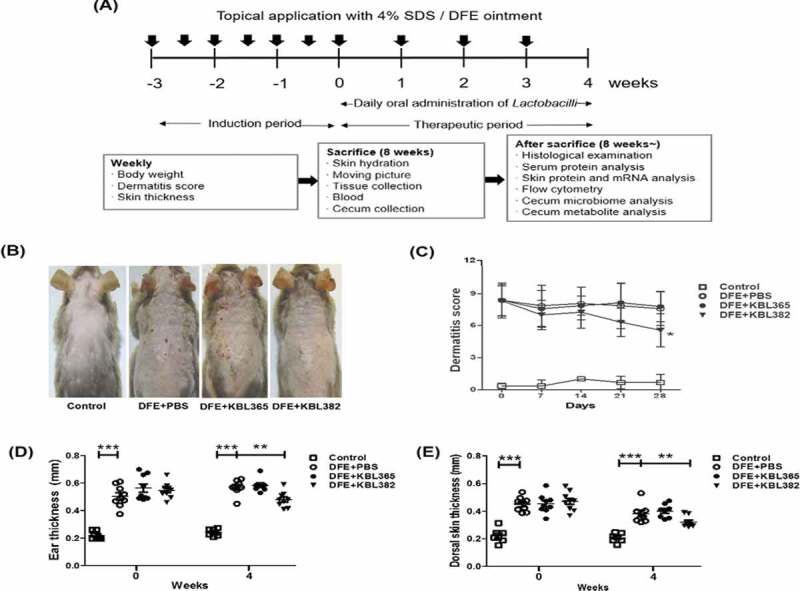
Symptom changes with oral administration of *L. rhamnosus* KBL365 or *L. paracasei* KBL382 in mice with *Dermatophagoides farinae* extract (DFE)-induced atopic dermatitis (AD)-like symptoms. (A) Experimental design. The mice were treated with DFE for 7 weeks. After 3 weeks from the initial DFE application, mice were fed KBL382 or KBL365 for 4 weeks. Body weight and dermatitis scores were measured at 1-week intervals. (B) Photographs of DFE-induced dermatitis in NC/Nga mice were taken on d 49. Four groups of mice (*N =* 7–9) were treated with (1) PBS (Control), (2) DFE+PBS, (3) DFE+KBL365, and (4) DFE+KBL382. (C) Dermatitis scores were evaluated once a week for 4 weeks after bacterial administration. (D) Ear and (E) dorsal skin thickness were measured once a week for 4 weeks. Statistical analyses were performed using the Mann–Whitney U-test for comparison with DFE+PBS mice (*N* = 7–9 mice per group). Error bars represent SEM. * *P*< .05; ** *P*< .01; *** *P*< .001.

The overall experimental procedure is illustrated in Figure 1A. When the severity of dermatitis was analyzed visually from photographs, we found that oral administration of KBL382 ameliorated the development of AD-like lesions compared to DFE+PBS mice, whereas no difference was observed between KBL365-treated and DFE+PBS mice (Figure 1B). On week 4, oral administration of KBL382 significantly suppressed the development of dermatitis scores compared to DFE+PBS mice. However, no difference was observed between KBL365-treated mice and DFE+PBS mice (Figure 1C). We also measured the thickness of ear and dorsal skin from 0 to 4 weeks. On week 4, the thickness of KBL365-treated, DFE+PBS mice were similar. By contrast, that of KBL382-treated mice was significantly reduced compared to KBL365-treated or DFE+PBS mice (Figure 1D and E). Furthermore, AD-like symptoms induced by 2, 4-dinitrochlorobenzene (DNCB) were also ameliorated in KBL382-treated mice only (Figure S1A-D).

### KBL382 treatment enhances skin function in dorsal skin lesions and induces immune homeostasis including IgE activity in AD

**Figure 2.**
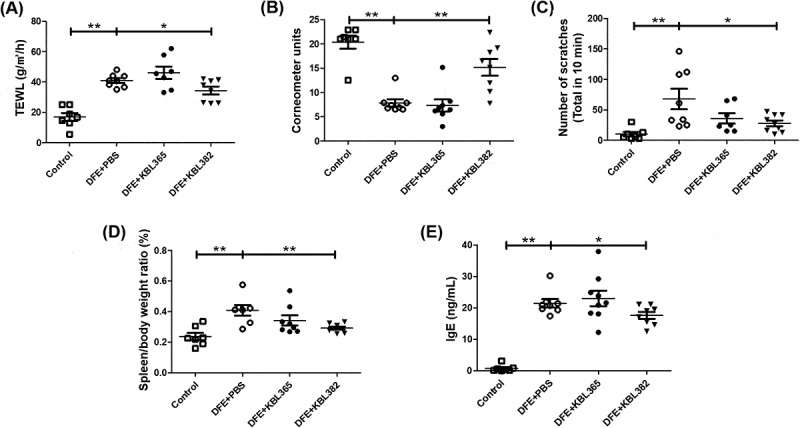
Effects of oral administration of *L. rhamnosus* KBL365 or *L. paracasei* KBL382 on epidermal hydration, scratching behavior, and overall immune response in AD mice. To evaluate epidermal hydration, (A) transepidermal water loss (TEWL) and (B) corneometer units were measured. (C) Scratching behavior was observed for 10 min on d 49. (D) The ratio of spleen-to-body weight was calculated on d 49 after sacrifice. (E) The concentration of immunoglobulin (Ig)E in collected serum on d 49 was determined using an ELISA kit. Statistical analyses were performed using the Mann–Whitney U-test for comparison with DFE+PBS mice (*N* = 7–9 mice per group). Error bars represent SEM. * *P*< .05; ** *P*< .01.

We measured TEWL and corneometer units, which indicated hydration and moisture in skin, as well as observing scratching behavior, the ratio of spleen-to-body weight, and IgE levels. Although the TEWL was similar between KBL365-treated mice and DFE+PBS mice, that of KBL382-treated mice was significantly lower than that of DFE+PBS mice, in accordance with the symptoms observed *in vivo* (Figure 2A). Oral administration of KBL382 significantly increased corneometer unit compared to DFE+PBS mice. On the other hand, oral administration of KBL365 was not related to skin moisture (Figure 2B).

The scratching behavior of KBL365-treated mice tended to be less severe than that of DFE+PBS mice, but no significant differences were observed. By contrast, oral administration of KBL382 significantly reduced the frequency of scratching behavior (Figure 2C). In addition, we compared the weight ratio of the spleen among groups to assess the immune response, in light of previous research showing that an enlarged spleen indicates abnormal immune system function in AD.^30^ Oral administration of KBL382 led to a significantly lower spleen-to-body weight ratio compared to DFE+PBS mice (Figure 2D). We also investigated the ameliorative effects of KBL382 on serum levels of IgE. As observed in DFE+PBS mice, AD was accompanied by a marked increase in IgE concentrations (Figure 2E). Compared to DFE+PBS mice, the IgE concentrations of KBL382-treated mice were significantly lower. Meanwhile, KBL365-treated mice were comparable to DFE+PBS mice in terms of IgE (Figure 2E). In DNCB-induced model, the spleen-to-body ratio and serum levels of IgE in KBL382-treated mice were also significantly lower than PBS-treated mice (Figure S2A-B).

### KBL382 treatment reduces infiltration of eosinophils and mast cells into dorsal skin lesions

**Figure 3.**
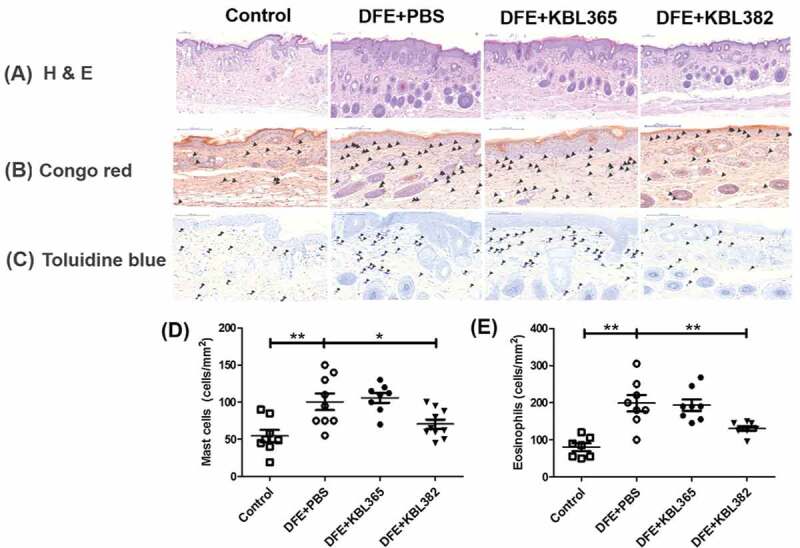
Effects of oral administration of *L. rhamnosus* KBL365 or *L. paracasei* KBL382 on dorsal skin inflammation in AD mice. Dorsal skin was excised, fixed with 10% formalin, embedded in paraffin, and stained with (A) hematoxylin and eosin, (B) Congo red, and (C) toluidine blue. Arrowheads on the images indicate mast cells and eosinophils, and their numbers are presented in (D) and (E), respectively. Statistical analyses were performed using the Mann–Whitney U-test for comparison with DFE+PBS mice (*N* = 7–9 mice per group). Error bars represent SEM. * *P*< .05; ** *P* < .01.

DFE+PBS mice showed severe AD-like lesions and hyperkeratosis of the skin. However, administration of KBL382 led to a decrease in epidermal thickness in skin with AD (Figure 3A). To evaluate skin lesions, eosinophils and mast cells were stained with Congo red and toluidine blue, respectively. The numbers of both eosinophils and mast cells were significantly reduced in KBL382-treated mice compared to DFE+PBS mice, while there were no significant differences between KBL365-treated mice and DFE+PBS mice (Figure 3B–E). Also, administration of KBL382 led decrease in epidermal thickness of skin lesion in mice with DNCB-induced AD (Figure S1E).

### KBL382 treatment modulates TSLP, pro-inflammatory chemokines, and cytokines in dorsal skin lesions

**Figure 4.**
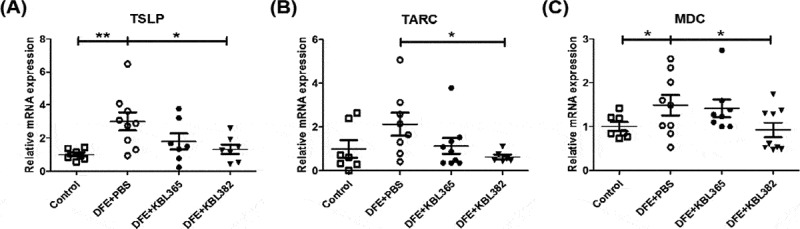
Effects of oral administration of *L. rhamnosus* KBL365 or *L. paracasei* KBL382 on innate cytokine and chemokine expression in the skin of AD mice. (A) Thymic stromal lymphopoietin (TSLP), (B) thymus and activation regulated chemokine (TARC) and (C) macrophage-derived chemokine (MDC) were analyzed through real-time PCR. Statistical analyses were performed using the Mann–Whitney U-test for comparison with DFE+PBS mice (*N=* 7–9 mice per group). Error bars represent SEM. * *P*< .05; ** *P*< .01.

**Figure 5.**
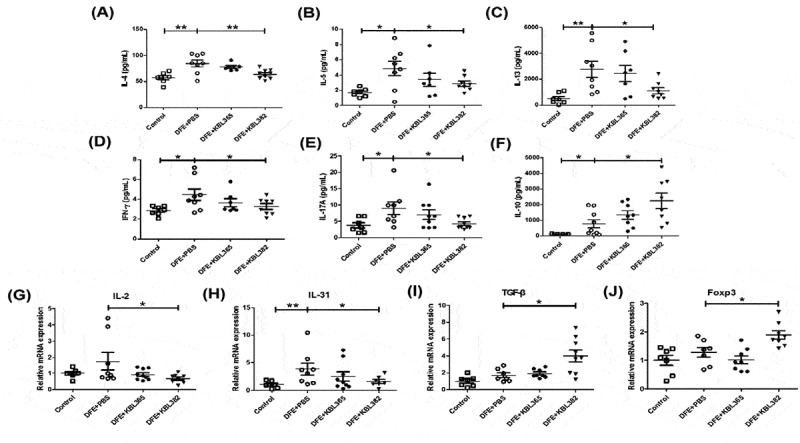
Effects of oral administration of *L. rhamnosus* KBL365 or *L. paracasei* KBL382 on Th1, Th2, Th17, and anti-inflammatory cytokine production in the skin of AD mice. Protein levels of the T helper (Th)2-type cytokines (A) interleukin (IL)-4, (B) IL-5, and (C) IL-13, Th1-type cytokine (D) IFN-γ, Th17-type cytokine (E) IL-17A, and anti-inflammatory cytokine (F) IL-10 were measured using a multiplex magnetic Luminex kit. Total RNA was extracted from skin and mRNA expression levels of cytokine genes related to Th1-type (G) IL-2, Th2-type (H) IL-31, anti-inflammatory cytokine (I) TGF-β and regulatory T cell (Treg) marker (J) Foxp3 were evaluated using real-time PCR. Statistical analyses were performed using the Mann–Whitney U-test for comparison with DFE+PBS mice (*N=* 7–9 mice per group). Error bars represent SEM. * *P*< .05; ** *P*< .01.

The levels of TSLP, TARC, and MDC were significantly reduced in KBL382-treated mice compared to DFE+PBS mice (Figure 4A–C). However, they were not significantly different in KBL365-treated mice compared to DFE+PBS mice (Figure 4A–C). We further evaluated the immune response related to T cells based on protein and mRNA levels in the skin. The levels of Th1- (IL-2 and IFN-γ), Th2- (IL-4, IL-5, IL-13, and IL-31), and Th17- (IL-17A) were significantly suppressed in KBL382-treated mice, whereas there were no significant differences between KBL365-treated and DFE+PBS mice (Figure 5A–E, G, and H). By contrast, significantly elevated levels of IL-10, transforming growth factor (TGF)-β and Foxp3 expression were observed in KBL382-treated mice compared to DFE+PBS mice. Oral administration of KBL365 also increased the IL-10 level and TGF-β expression compared to DFE+PBS mice, but this effect was not significant (figure 5F and I-J). IL-4, IL-5, and IL-13 were significantly decreased, and IL-10 and Foxp3 were clearly elevated in the DNCB-induced AD mice with KBL382 treatment (Figure S2C-E, H, and I).

### KBL382 treatment increases the Treg cells population in the mesenteric lymph node (MLN)

**Figure 6.**
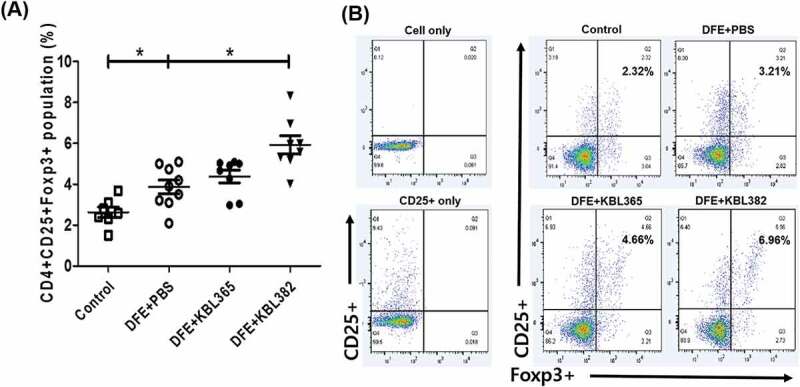
Effects of oral administration of *L. rhamnosus* KBL365 or *L. paracasei* KBL382 on Treg in mesenteric lymph nodes (MLNs) of AD mice. The MLN cells isolated from NC/Ng mice in Control, DFE+PBS, KBL365-treated, and KBL382-treated mice. (A and B) The number of CD4+ CD25+ Foxp3+ cells was calculated based on the percentage of total MLN cell counts. Statistical analyses were performed using the Mann–Whitney U-test for comparison with DFE+PBS mice (*N=* 7–9 mice per group). Error bars represent SEM. * *P*< .05.

We assessed whether oral administration of KBL365 or KBL382 leads to an increase in Treg cells in the MLN. The proportions of CD4+ CD25+ Foxp3+ Tregs cells in KBL382-treated mice were significantly elevated compared to that of DFE+PBS mice. Oral administration of KBL382 promoted the generation of Tregs in MLN to modulate the immune response. Administration of KBL365 also increased the CD4+ CD25+ Foxp3+ Tregs cells compared to DFE+PBS mice, but this effect was not significant (Figure 6A and B).

### KBL365 or KBL382 treatment alters the cecum microbiota in AD

**Figure 7.**
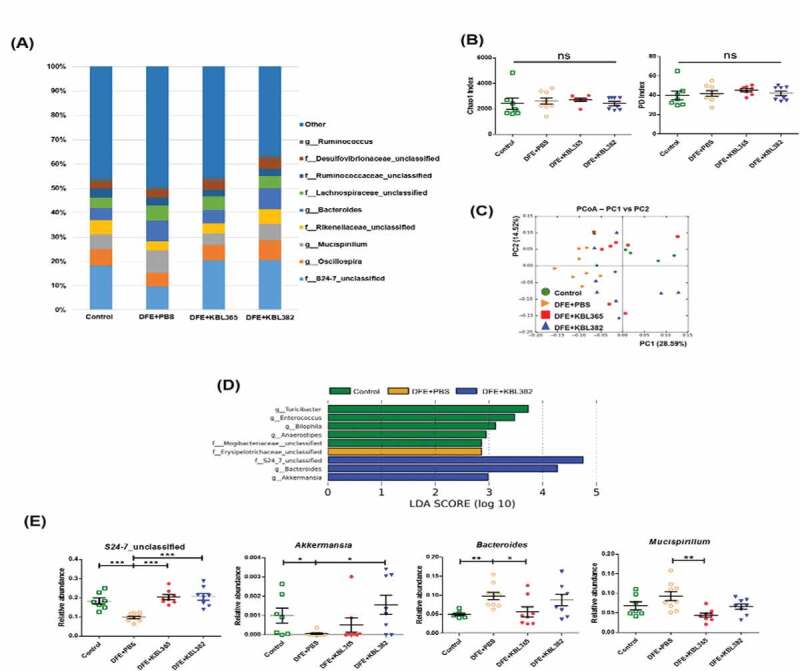
Effects of oral administration of *L. rhamnosus* KBL365 or *L. paracasei* KBL382 on the cecal microbiota of AD mice. Microbiome analyses were carried out using cecal samples from 7–9 mice per group. (A) Average relative abundances of taxa at the genus level. (B) Rarefaction plots based on the chao1 and phylogenic diversity (PD) indexes. (C) Principal component analyses of cecal microbiota structure based on weighted UniFrac distance. (D) Significantly different taxa among Control (green), DFE+PBS (yellow), and DFE+KBL382 (blue) samples, as determined using LEfSe analyses (LDA-score >2.5). (E) Comparison of relative abundances of significantly different microbial taxa at the genus level. Statistical analyses were performed using the Mann–Whitney U-test with no false discovery rate (FDR)-correction for comparison with DFE+PBS mice (*N=* 7–9 mice per group). Error bars represent SEM. * *P*< .05; ** *P*< .01; *** *P*< .001.

At the genus level, the bacterial microbiota of Control mice was mainly composed of *S24–7_unclassified* (18.2%) followed by *Bacteroides* (4.8%), while DFE+PBS mice reduced abundances of *S24–7_unclassified* (9.7%) and increased *Bacteroides* (8.5%). Mice administered the two strains showed a microbiota similar to Control mice, as *S24–7_unclassified* (20.2%) and *Bacteroides* (5.6%) were common in KBL365-treated mice and these taxa (20.5% and 8.7%, respectively) were also abundant in KBL382-treated mice (Figure 7A). Chao1 and phylogenetic diversity (PD), indicating bacterial alpha diversity, revealed no differences among the four groups (Figure 7B). Principle coordinate analyses (PCoA) of the weighted UniFrac distances indicated that bacterial composition (beta diversity) in the gut separated distinctly in groups of KBL365 or KBL382 administration compared to the group with DFE application only (Figure 7C). In further taxonomic analyses, the two *Lactobacillus*-administration groups had significantly higher levels of *S24–7_unclassified* compared to DFE+PBS mice. KBL365 significantly decreased *Mucispirillum* and *Bacteroides*, for a composition similar to Control mice. KBL382 significantly increased the relative abundance of *Akkermansia* compared to DFE+PBS mice, with much higher levels than that in control mice (Figure 7D and E).

### KBL365 or KBL382 treatment regulates metabolites in cecum

**Figure 8.**
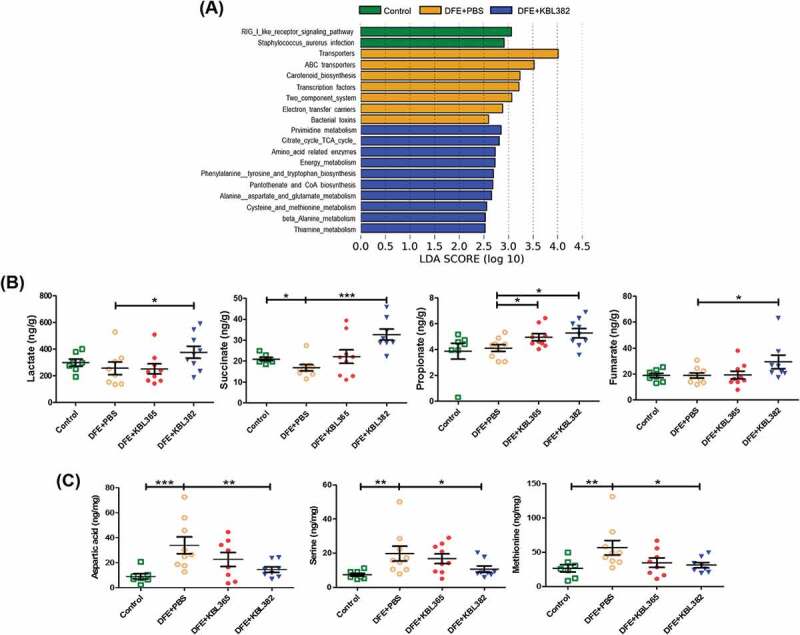
Effects of oral administration of *L. rhamnosus* KBL365 or *L. paracasei* KBL382 on cecal metabolites of AD mice. (A) Significant changes in functional profiles were suggested based on phylogenetic investigations of communities through reconstruction of unobserved data and LEfSe analyses with the Kyoto Encyclopedia of Genes and Genomes (KEGG) pathway database (LDA-score > 2.5). (B) Analyses of nonvolatile acids and short-chain fatty acid using gas chromatography. (C) Analyses of amino acids using ultra-performance liquid chromatography. Statistical analyses were performed using the Mann–Whitney U-test with no FDR correction for comparison with DFE+PBS mice (*N=* 7–9 mice per group). Error bars represent SEM. * *P*< .05; ** *P*< .01; *** *P*< .001.

DFE+PBS mice exhibited increases in environmental information processes, genetic information processes, and cellular processes (Figure 8A). By contrast, KBL382-treated mice showed involvement of pathways such as metabolism and biosynthesis. Next, we investigated amino acids, nonvolatile acids, and short-chain fatty acid (SCFA) such as propionate in cecum. KBL382-treated mice showed significant increases in the concentrations of both nonvolatile acids such as lactate, succinate, and fumarate, and SCFA such as propionate compared to DFE+PBS mice (Figure 8B). On the other hand, KBL382-treated mice showed significant decreases of aspartic acid, serine, and methionine compared to DFE+PBS mice (Figure 8C).

## Discussion

Our study demonstrated that the administration of specific *Lactobacillus* strain could significantly alleviate the symptoms of AD via modulation of the immune response and gut microbiota. We used DFE and DNCB, which causes AD-like skin lesions, in an NC/Nga mouse model. External exposure to epicutaneous allergens such as DFE and DNCB can induce localized allergic inflammation in the skin and systemic sensitization to a specific allergen.^27^ The symptoms of skin lesions were categorized based on edema, hemorrhage, erosion, dryness, and were significantly alleviated in the AD mouse model through administration of KBL382. Lichenification, which is indicated by ear and dermal thickness, was reduced in KBL382-treated mice compared to DFE+PBS and DNCB+PBS mice (Figure 1D, E, 3A, and S1D-E).^31^ KBL382-treated mice had a significantly lower TEWL and higher corneometer units than DFE+PBS mice, indicating fewer defects in the intercellular lipid bilayers of the stratum corneum (Figure 2A and B).^14^ Furthermore, scratching behavior increased during the progression of AD, suggesting increases in IgE and IL-31 levels and the number of mast cells.^32^ Scratching behavior frequency was significantly lower in KBL382-treated mice than in DFE+PBS mice due to the reduced inflammation response (Figure 2C). KBL382 also decreased the production of IgE, and thus controlled clinical symptoms related to AD (Figure 2E and S2B). A decrease in serum concentrations of IgE suppresses the activation of mast cells and eosinophils, which express high-affinity receptors for IgE on their surface to induce degranulation of inflammatory mediators such as histamine and tryptase.^23^ KBL382 significantly suppressed the infiltration of both eosinophils and mast cells into affected skin lesions (Figure 3B and C). Exposure to allergens on atopic skin increases the expression of TSLP in epithelial cells, particularly keratinocytes. TSLP is a potent activator of DCs and induces production of the Th2-attracting chemokines TARC and MDC. TSLP-activated DCs prime naïve Th cells to produce the proallergic cytokines IL-4, IL-5, and IL-13, while downregulating IL-10.^33^ The expression levels of TSLP, MDC, and TARC were elevated in DFE+PBS mice but reduced in KBL382-treated mice, which may suppress differentiation of T cells into Th2 cells and the secretion of Th2-type cytokines (Figure 4).

As Th1, Th2, and Th17 responses are involved in the pathogenesis of AD,^8,10,14^ oral administration of KBL382 modulated the adaptive immune response, leading to suppression of Th1 (IL-2 and IFN-γ), Th2 (IL-4, IL-5, IL-13, and IL-31), and Th17 (IL-17A) cytokines (Figure 5A-E, G-H, and S2C-E). Ingested probiotics are recognized by pattern recognition receptors, such as Toll-like receptors (TLRs) on DCs, and are essential to immunological homeostasis in the gut. DCs can directly present antigens from probiotics to the MLN and can interact with T and B cells to maintain noninflammatory immune responses. During this process, DCs play an important role in converting CD4+ Foxp3-T cells into CD4+ Foxp3 + T cells in MLNs.^34,35^ CD4+ Foxp3+ Treg cells produce IL-10 and TGF-β, which suppress T cell polarization into Th2 cells, leading to the production of IL-4, IL-5, and IL-13 and inhibition of the activation of T cells, monocytes, and DCs.^23,34^ Some probiotics increase the abundance of Treg cells, which mediate immune suppression and attenuate skin symptoms of AD.^26^ Our results also showed that KBL382 significantly promoted the Treg cells population compared to DFE+PBS mice, downregulating Th2 cells and inhibiting the progression of inflammation (Figure 5J, 6 and S2I). Gut microbiota interact dynamically with other microorganisms and hosts and it has become increasingly apparent that they are crucial to host health.^36^ Interestingly, although alpha diversity was similar among the four groups tested, the beta diversity of gut microbiota differed distinctly (Figure 7B and C). The proportions of bacteria at the genus level differed depending on DFE treatment and administration of the two strains. In accordance with previous study gut microbiota was altered in the progression of colitis, with a reduced relative abundance of *S24–7_unclassified* and increased *Bacteroides* and *Mucispirillum*.^37^ On the other hand, we observed an increased abundance of *S24–7_unclassified* with administration of KBL365 or KBL382, and decreased abundance of *Bacteroides* and *Mucispirillum* with administration of KBL365 (Figure 7A, D, and E). The proportion of *Akkermansia* was significantly increased with administration of KBL382 in AD-induced mice (Figure 7D and E). Previous study has shown that the relative abundance of *Akkermansia* is significantly reduced in IBD patients.^38^ *Akkermansia* increases anti-inflammatory activity by inducing Treg cells in adipose tissue, leading to attenuation of high-fat-diet-induced metabolic syndrome.^39^ In addition, *Akkermansia* stimulates IL-10 production, playing an immunological role in homeostasis and taking on a barrier function in the gut mucosa.^40^ Therefore, the further longitudinal study to elucidate the effects of KBL382 treatment in the abundances of beneficial microorganisms including *Akkermansia* should be performed. The compositional changes in specific microorganisms in the gut and their functional roles in the phenotypic changes observed in AD should be evaluated.

To investigate the functional roles of the administered *Lactobacillus* strains, we used function prediction to characterize gut microbiota using the Kyoto Encyclopedia of Genes and Genomes (KEGG) pathway database and measured metabolites in cecum. Gut microbiota is important to produce nonvolatile acids such as lactate, succinate, and fumarate which serve as pivotal intermediates that are degraded into SCFAs.^41^ KBL382-treated mice had reduced amino acid levels but elevated levels of nonvolatile acids and propionate compared to DFE+PBS mice. These findings were consistent with KEGG analyses showing that administration of KBL382 resulted in increased levels of genes associated with metabolism. Moreover, KBL382 administration can affect the amino acids and SCFAs production (Figure 8A–C). Further study to clarify the microbiota-derived SCFAs related to KBL382 administration should be performed.

Previous studies suggested that *L. rhamnosus* showed the therapeutic effects for AD.^42–44^ Especially, *L. rhamnosus* GG is widely reported strain for AD prevention and treatment.^43,44^ Therefore, in this study, we used *L. rhamnosus* KBL365, isolated from healthy Korean feces, to confirm the effects of *L. paracasei* on AD. Although some *L. rhamnosus* strains suppress the progression of symptoms^43^ and show therapeutic benefits in infants with AD,^44^ KBL365 administration in our study did not suppress AD symptom progression due to differences in the adaptive immune response and biological functions of these strains, even within the same species. This is not surprising because strain specificity of particular functions is commonly observed in bacteria. Compared to more complex organisms, the same species of microorganisms can have a lower percentage of common genes, which can cause different functional capabilities of different strains within a genus or even species.^45^ The genes and functions of KBL365 and KBL382 should be further identified and compared, along with those of other strains, in the future.

In conclusion, administration of KBL382 alleviates AD by reducing the severity of clinical symptoms by increasing the immunosuppressive response and changing the metabolic functions of gut microbiota. Our results suggest that KBL382 is a promising candidate for therapeutic treatment to reduce AD symptoms.

## Materials and Methods

### *Isolation and preparation of* L. rhamnosus *KBL365 and* L. paracasei *KBL382*

*L. rhamnosus* KBL365 and *L. paracasei* KBL382 were isolated from the feces of healthy Koreans, as described in a previous study.^46^ Briefly, KBL365 and KBL382 strains were cultured in Lactobacilli MRS Agar (BD Difco, Sparks, MD, USA) supplemented with 0.05% L-cysteine-hydrochloride at 37°C under anaerobic conditions maintained with an Anaeropack (Mitsubishi Gas Chemical Company, Inc., Tokyo, Japan) for 24 h. The cells were harvested through centrifugation (1,200 × g) and washed twice with phosphate-buffered saline (PBS) prior to administration to mice. Based on decreases in levels of Th1, Th2, and Th17-type cytokines and an increase in the anti-inflammatory cytokine IL-10 in peripheral blood mononuclear cells *in vitro*,^46^ we selected KBL382 for our AD-like experiment.

### Experimental atopic dermatitis

Male 5-week-old NC/Nga mice were purchased from Central Lab Animals Incorporated (Seoul, Korea). All animal experimental procedures were approved by the Institutional Animal Care and Use Committee (IACUC: SNU–160928–1–1 and SNU-190919-3-1) of Seoul National University, Korea. The DFE and DNCB (Sigma-Aldrich Corp, St. Louis, MO, USA)-induced *in vivo* mouse AD model was performed as previously described with minor modifications.^5,31^ For DFE-induced model experiment, four groups of mice (*N* = 7–9) were treated with (1) PBS (Control), (2) DFE+PBS, (3) DFE+KBL365, and (4) DFE+KBL382. After removing hair from the ears and dorsal region using electric clippers and hair removal cream, all groups except Control were applied with 150 µL 4% sodium dodecyl sulfate for 3 h to disrupt the skin barrier. Then, they were treated with 100 mg Biostir AD (Biostir, Hiroshima, Japan), an ointment containing DFE extract, once or twice per week for 7 weeks to induce AD. After 3 weeks from the beginning of the experiment, the DFE+KBL365 and DFE+KBL382 groups were administered suspensions of KBL365 and KBL382 (1 × 10^9^ CFU each) in 200 μL PBS and Control and DFE+PBS mice were administered PBS daily via oral gavage for 4 weeks (Figure 1A). For DNCB-induced model experiment, three groups of mice (*N =* 6–7) were treated with (1) PBS (Control), (2) DNCB+PBS, and (3) DNCB+KBL382. After removing hair from the ears and dorsal region using electric clippers and hair removal cream, all groups except Control were applied with 200 µL of 1% DNCB dissolved in a vehicle (acetone:olive oil = 3:1) and 15 µL to induce skin legion on d 1 and 4. One week later, 0.4% DNCB was treated to challenge the dorsal skin (200 µL) and ears (15 µL) three times a week for 2 weeks. After 1 week from the beginning of the experiment, the DNCB+KBL382 group was administered suspensions of KBL382 (1 × 10^9^ CFU each) prepared in the same as the DFE-induced AD model experiment daily via oral gavage for 2 weeks (Figure S1A). Then, the mice were sacrificed and further analyzed.

### Assessment of skin lesions and clinical dermatitis severity score

NC/Nga mice were anesthetized with isoflurane, and pictures of the skin were taken weekly until the end of the experiment. The severity of dermatitis was evaluated once a week by scoring the symptoms visible on the ear, neck, and dorsal skin. The severity of dermatitis was assessed based on the four aspects of erythema/hemorrhage, scarring/dryness, edema, and excoriation/erosion on a scale of 0 (none), 1 (mild), 2 (moderate), and 3 (severe). A total dermatitis score was evaluated as the sum of the scores for each aspect, for a maximum score of 12.^31^

### Measurement of TEWL and stratum corneum hydration

On the last day of the experiment, TEWL and skin hydration were determined with a Tewameter TM300 and Corneometer CM 825 (Courage and Khazaka, Cologne, Germany), respectively, applied to the dorsal skin under controlled conditions of 21–22°C and 50–55% humidity.^47^ The probe was placed on the skin and stabilized for approximately 30 s, and then the measurement was recorded. This measurement procedure was repeated five times in the same area at the same pressure.

### Scratching behavior score

Scratching behavior was observed, as described previously.^48^ Briefly, the mice were placed individually in acrylic cases composed of three equal cells for at least 1 h for acclimation. Scratching behavior was evaluated by measuring the number of scratches and time that mice spent scratching their nose, ears, and dorsal skin for 10 min on the day before sacrifice. As mice make several rapid scratching movements over a period of about 1 s, a series of such movements was counted as one bout of scratching.^49^

### Measurement of total IgE

Blood was obtained from the eye blood vessel of each mouse. Serum was separated through centrifugation at 1,200 × g for 15 min at 4°C. Total serum concentrations of IgE were measured using an IgE ELISA kit (Komabiotech, Seoul, Korea) according to the manufacturer’s instructions.

### Measurement of cytokines and chemokines

To measure cytokine production at the protein level, frozen skin tissues were weighed and homogenized with radioimmunoprecipitation assay buffer and protease inhibitors (Thermo Fisher Scientific, Inc., Waltham, MA, USA). The homogenates were centrifuged at 12,000 × g for 10 min at 4°C, and their supernatants were collected. Levels of IFN-γ, IL-4, IL-5, IL-13, IL-10, and IL-17 were measured in the skin lysates using a customized multiplex magnetic Luminex kit (R&D Systems, Minneapolis, MN, USA). Expression of Foxp3, IL-2, IL-4, IL-5, IL-6, IL-10, IL-31, MDC, TARC, TGF-β, TNF-α, and TSLP in mouse skin tissue at mRNA level was measured using real-time PCR. RNA was isolated from skin using an Easy-spin Total RNA Extraction Kit (Intron, Seoul, Korea) and cDNA was synthesized using the High-Capacity RNA to cDNA Kit (Thermo Fisher Scientific). Real-time PCR was performed in a Rotor-Gene Q (Qiagen, Hilden, Germany) thermal cycler using the QuantiTect SYBR Green PCR kit (Qiagen) with the primers (0.01 mM) listed in Table S1. Each PCR consisted of denaturation at 95°C for 10 min, followed by 40 cycles of 95°C for 5 s and 60°C for 10 s. The relative expression level of the target gene was calculated using the 2^–ΔΔCT^ method, with normalization to the expression level of the reference gene hypoxanthine-guanine phosphoribosyl transferase.^50^

### Histological examination

Dorsal skin tissues were fixed in 10% formaldehyde. This tissue was stained with hematoxylin and eosin to evaluate neutrophil infiltration and with Congo red and toluidine blue to detect eosinophil and mast cell infiltration, respectively. The numbers of eosinophils and mast cells were counted using Pannoramic Viewer software (3DHISTECH, Ltd., Budapest, Hungary).

### Flow cytometric analyses

Flow cytometric analyses were carried out as described previously.^46^ Briefly, isolated MLN cells were stained with Fixable Viability Stain 510 (FVS510; BD bioscience, Franklin Lakes, NJ, USA) for live cells, as well as CD3+ fluorescein isothiocyanate (145–2 C11; BD bioscience), CD4+ Percep-Cyanine5.5 (RM4–5; BD bioscience) and CD25+ phycoerythrin (PC61; BD Bioscience) for cell surface staining after blocking with FcγR and permeabilization with fixation/permeabilization buffer (Ebioscience, San Diego, CA, USA) and were also treated with Foxp3+ Alexa Fluor 647 (MF23; BD Bioscience) for intracellular staining. IgG isotypes were used as a control in all flow cytometry experiments. The CD4+ CD25+ Foxp3+ Treg population was analyzed using a BD FACSVerse™ Flow Cytometer (BD Bioscience).

### Intestinal microbiota analyses

Intestinal microbiota was observed as described previously.^46^ Briefly, the V3-V4 region of the 16S rRNA gene in total genomic DNA collected from cecum was amplified using the barcoded primers 341F and 805R. PCR amplicons were purified using the QIAquick PCR Purification Kit (Qiagen) and sequenced on the MiSeq platform (Illumina, Inc., San Diego, CA, USA).^51,52^ Sequence data were processed using the Quantitative Insights into Microbial Ecology 1.8.0 (QIIME) pipeline. The sequences were clustered into operational taxonomic units (OTUs) at the 97% identity level using the Greengenes database (ver. 13_5). Alpha diversity indexes such as chao1 and PD were estimated to identify significant differences among groups. Phylogenetic beta diversity was measured using the UniFrac distance between samples and visualized based on weighted PCoAs. Linear discriminant analyses (LDAs) of effect size (LEfSe) were performed (http://huttenhower.org/galaxy) to identify significantly different phylotypes within the experimental groups. In addition, phylogenetic investigation of the communities was performed by reconstructing unobserved data to identify functional genes in the sampled microbial community based on data in the KEGG pathway database (GenomeNet; https://www.genome.jp/kegg/pathway.html), as described previously.^53^

### Measurement of nonvolatile acids and short-chain fatty acid

Cecum was homogenized with distilled water and centrifuged at 13,000 × g for 5 min. Then, the supernatant was extracted and mixed with an internal standard (benzoic acid for nonvolatile acids and 1% 2-methylpentanoic acid for volatile acids). The supernatant was added to the extraction solvent (chloroform for nonvolatile acids and ethyl ether for volatile acids) and centrifuged at 13,000 × g for 5 min. The organic layer was transferred to a vial (Agilent Technologies, Santa Clara, CA, USA) and measured through gas chromatography. Nitrogen was used as the carrier gas. The oven temperature was set to 170°C. The flame ionization detector and injection port were set to 225°C. The retention times and peak areas of the standard mix were used as references for unknown samples.^54^

### Amino acid analyses using ultra-performance liquid chromatography (UPLC)

Amino acids were analyzed as described previously.^46^ Cecum extracts were transferred to an autosampler vial and derivatized. After incubation for 10 min at 55°C, the derivatized samples were analyzed using Acquity UPLC (Waters Corporation) with a SYNAPT G2-Si mass spectrometer (Waters Corporation). The mass acquisition mode Tof-MRM and positive electrospray ionization mode were used. Capillary voltage was set to 1.5 kV. The sampling cone voltage varied from 20 V to 60 V among amino acids. The desolvation gas and cone gas flow rates were set to 600 L/h and 50 L/h, respectively. The desolvation temperature was 250°C. Amino acids were confirmed through alignment to the analytical standard mixture and internal standard. Data acquisition and quantification were carried out using MassLynx software 4.1 (Waters Corporation).^55^

### Statistical analyses

Data are expressed as mean ± standard error of the mean (SEM). GraphPad Prism 5.04 (GraphPad Software, Inc., La Jolla, CA, USA) was used to visualize and analyze data using the Mann–Whitney U test. When appropriate, false discovery rate (FDR)-correction was not applied in data presentation. Statistical significance is denoted as * *P*< .05, ** *P*< .01, *** *P*< .001.

## Supplementary Material

Supplemental Material

## References

[1] Nutten S. Atopic dermatitis: global epidemiology and risk factors. Ann Nutr Metab. 2015;1:8–16. doi:10.1159/000370220. 25925336

[2] Furue M, Chiba T, Tsuji G, Ulzii D, Kido-Nakahara M, Nakahara T, Kadono T. Atopic dermatitis: immune deviation, barrier dysfunction, IgE autoreactivity and new therapies. Allergol Int. 2017;66(3):398–403. doi:10.1016/j.alit.2016.12.002. 28057434

[3] Bieber T. Atopic dermatitis. N Engl J Med. 2008;22(2):125–137. doi:10.5021/ad.2010.22.2.125.

[4] Kim JY, Park BK, Park HJ, Park YH, Kim BO, Pyo S. Atopic dermatitis-mitigating effects of new *Lactobacillus* strain, *Lactobacillus sakei* probio 65 isolated from Kimchi. J Appl Microbiol. 2013;115(2):517–526. doi:10.1111/jam.12229. 23607518

[5] Karki R, Jung M-A, Kim K-J, Kim D-W. Inhibitory effect of *Nelumbo nucifera* (Gaertn.) on the development of atopic dermatitis-like skin lesions in NC/Nga mice. Evid Based Complement Alternat Med. 2012;2012:7. doi:10.1155/2012/153568. PMC3291163 22454654

[6] Gandhi NA, Bennett BL, Graham NMH, Pirozzi G, Stahl N, Yancopoulos GD. Targeting key proximal drivers of type 2 inflammation in disease. Nat Rev Drug Discov. 2016;15(1):35–50. doi:10.1038/nrd4624. 26471366

[7] Nakazato J, Kishida M, Kuroiwa R, Fujiwara J, Shimoda M, Shinomiya N. Serum levels of Th2 chemokines, CCL17, CCL22, and CCL27, were the important markers of severity in infantile atopic dermatitis. Pediatr Allergy Immunol. 2008;19(7):605–613. doi:10.1111/j.1399-3038.2007.00692.x. 18266834

[8] Spergel JM, Mizoguchi E, Oettgen H, Bhan AK, Geha RS, Herz U, Bunikowski R, Renz H, Leung D, Leiferman K, et al. Roles of TH1 and TH2 cytokines in a murine model of allergic dermatitis. J Clin Invest. 1999;103(8):1103–1111. doi:10.1172/JCI5669. 10207161 PMC408277

[9] Matsuda H, Watanabe N, Geba GP, Sperl J, Tsudzuki M, Hiroi J, Matsumoto M, Ushio H, Saito S, Askenase PW, et al. Development of atopic dermatitis-like skin lesion with IgE hyperproduction in NC/Nga mice. Int Immunol. 1997;9(3):461–466. doi:10.1093/intimm/9.3.461. 9088984

[10] Takeuchi S, Yasukawa F, Furue M, Katz SI. Collared mice: A model to assess the effects of scratching. J Dermatol Sci. 2010;57(1):44. doi:10.1016/j.jdermsci.2009.09.008. 19896338 PMC2818307

[11] Zhao H, Li M, Wang L, Su Y, Fang H, Lin J, Mohabeer N, Li D. Angiotensin II induces TSLP via an AT1 receptor/NF-κB pathway, promoting Th17 differentiation. Cell Physiol Biochem. 2012;30(6):1383–1397. doi:10.1159/000343327. 23154814

[12] Gittler JK, Shemer A, Suárez-Fariñas M, Fuentes-Duculan J, Gulewicz KJ, Wang CQF, Mitsui H, Cardinale I, De Guzman Strong C, Krueger JG, et al. Progressive activation of TH2/TH22 cytokines and selective epidermal proteins characterizes acute and chronic atopic dermatitis. J Allergy Clin Immunol. 2012;130(6):1344–1354. doi:10.1016/j.jaci.2012.07.012. 22951056 PMC3991245

[13] Koga C, Kabashima K, Shiraishi N, Kobayashi M, Tokura Y. Possible pathogenic role of Th17 cells for atopic dermatitis. J Invest Dermatol. 2008;128(11):2625–2630. doi:10.1038/jid.2008.111. 18432274

[14] Boguniewicz M, Leung DYM. Atopic dermatitis: A disease of altered skin barrier and immune dysregulation. Immunol Rev. 2011;242(1):233–246. doi:10.1111/j.1600-065X.2011.01027.x. 21682749 PMC3122139

[15] Liu Y-J. Thymic stromal lymphopoietin: master switch for allergic inflammation. J Exp Med. 2006;203(2):269–273. doi:10.1084/jem.20051745. 16432252 PMC2118215

[16] Shimada Y, Takehara K, Sato S. Both Th2 and Th1 chemokines (TARC/CCL17, MDC/CCL22, and Mig/CXCL9) are elevated in sera from patients with atopic dermatitis. J Dermatol Sci. 2004;34(3):201–208. doi:10.1016/j.jdermsci.2004.01.001. 15113590

[17] Olszak T, An D, Zeissig S, Vera MP, Richter J, Franke A, Glickman JN, Siebert R, Baron RM, Kasper DL, et al. Microbial exposure during early life has persistent effects on natural killer T cell function. Science. 2012;336(6080):489–493. doi:10.1126/science.1219328. 22442383 PMC3437652

[18] Levy M, Blacher E, Elinav E. Microbiome, metabolites and host immunity. Curr Opin Microbiol. 2017;35:8–15. doi:10.1016/j.mib.2016.10.003. 27883933

[19] Pineiro M, Stanton C. Probiotic bacteria: legislative framework – requirements to evidence basis. J Nutr. 2007;137(3):850S–3S. doi:10.1093/jn/137.3.850S. 17311986

[20] Yeom M, Sur BJ, Park J, Cho SG, Lee B, Kim ST, Kim KS, Lee H, Hahm DH. Oral administration of *Lactobacillus casei* variety rhamnosus partially alleviates TMA-induced atopic dermatitis in mice through improving intestinal microbiota. J Appl Microbiol. 2015;119(2):560–570. doi:10.1111/jam.12844. 25968453

[21] Inoue R, Nishio A, Fukushima Y, Ushida K. Oral treatment with probiotic *Lactobacillus johnsonii* NCC533 (La1) for a specific part of the weaning period prevents the development of atopic dermatitis induced after maturation in model mice, NC/Nga. Br J Dermatol. 2007;156(3):499–509. doi:10.1111/j.1365-2133.2006.07695.x. 17300240

[22] Sunada Y, Nakamura S, Kamei C. Effect of *Lactobacillus acidophilus* strain L-55 on the development of atopic dermatitis-like skin lesions in NC/Nga mice. Int Immunopharmacol. 2008;8(13–14):1761–1766. doi:10.1016/j.intimp.2008.08.011. 18790088

[23] Won TJ, Kim B, Lim YT, Song DS, Park SY, Park ES, Lee DI, Hwang KW. Oral administration of *Lactobacillus* strains from Kimchi inhibits atopic dermatitis in NC/Nga mice. J Appl Microbiol. 2011;110(5):1195–1202. doi:10.1111/j.1365-2672.2011.04981.x. 21338447

[24] Sawada J, Morita H, Tanaka A, Salminen S, He F, Matsuda H. Ingestion of heat-treated *Lactobacillus rhamnosus* GG prevents development of atopic dermatitis in NC/Nga mice. Clin Exp Allergy. 2007;37(2):296–303. doi:10.1111/j.1365-2222.2006.02645.x. 17250703

[25] Tanaka A, Jung K, Benyacoub J, Prioult G, Okamoto N, Ohmori K, Blum S, Mercenier A, Matsuda H. Oral supplementation with *Lactobacillus rhamnosus* CGMCC 1.3724 prevents development of atopic dermatitis in NC/NgaTnd mice possibly by modulating local production of IFN-γ. Exp Dermatol. 2009;18(12):1022–1027. doi:10.1111/j.1600-0625.2009.00895.x. 19555432

[26] Shin JH, Chung MJ, Seo JG. A multistrain probiotic formulation attenuates skin symptoms of atopic dermatitis in a mouse model through the generation of CD4+Foxp3+ T cells. Food Nutr Res. 2016;60:32550. doi:10.3402/fnr.v60.32550. 27802847 PMC5090133

[27] Kim HJ, Kim YJ, Kang MJ, Seo JH, Kim HY, Jeong SK, Lee SH, Kim JM, Hong SJ. A novel mouse model of atopic dermatitis with epicutaneous allergen sensitization and the effect of *Lactobacillus rhamnosus*. Exp Dermatol. 2012;21(9):672–675. doi:10.1111/j.1600-0625.2012.01539.x. 22742655

[28] Van Baarlen P, Wells JM, Kleerebezem M. Regulation of intestinal homeostasis and immunity with probiotic lactobacilli. Trends Immunol. 2013;34(5):208–215. doi:10.1016/j.it.2013.01.005. 23485516

[29] Dupont AW, Dupont HL. The intestinal microbiota and chronic disorders of the gut. Nat Rev Gastroenterol Hepatol. 2011;8(9):523–531. doi:10.1038/nrgastro.2011.133. 21844910

[30] Han S-C, Kang G-J, Ko Y-J, Kang H-K, Moon S-W, Ann Y-S, Yoo E-S. Fermented fish oil suppresses T helper 1/2 cell response in a mouse model of atopic dermatitis via generation of CD4+CD25+Foxp3+ T cells. BMC Immunol. 2012;13:44. doi:10.1186/1471-2172-13-44. 22873180 PMC3537649

[31] Kang H, Lee CH, Kim JR, Kwon JY, Seo SG, Han JG, Kim BG, Kim JE, Lee KW. *Chlorella vulgaris* attenuates *Dermatophagoides farinae*-induced atopic dermatitis-like symptoms in NC/Nga mice. Int J Mol Sci. 2015;16(9):21021–21034. doi:10.3390/ijms160921021. 26404252 PMC4613239

[32] Takaoka A, Arai I, Sugimoto M, Honma Y, Futaki N, Nakamura A, Nakaike S. Involvement of IL-31 on scratching behavior in NC/Nga mice with atopic-like dermatitis. Exp Dermatol. 2006;15(3):161–167. doi:10.1111/j.1600-0625.2006.00405.x. 16480423

[33] Kim HJ, Kim HY, Lee SY, Seo JH, Lee E, Hong SJ. Clinical efficacy and mechanism of probiotics in allergic diseases. Korean J Pediatr. 2013;56(9):369–376. doi:10.3345/kjp.2013.56.9.369. 24223597 PMC3819679

[34] Lim SK, Kwon MS, Lee J, Oh YJ, Jang JY, Lee JH, Park HW, Nam YD, Seo MJ, Roh SW, et al. *Weissella cibaria* WIKIM28 ameliorates atopic dermatitis-like skin lesions by inducing tolerogenic dendritic cells and regulatory T cells in BALB/c mice. Sci Rep. 2017;7:40040. doi:10.1038/srep40040. 28067304 PMC5220369

[35] Kwon H-K, Lee C-G, So J-S, Chae C-S, Hwang J-S, Sahoo A, Nam JH, Rhee JH, Hwang K-C, Im S-H. Generation of regulatory dendritic cells and CD4+Foxp3+ T cells by probiotics administration suppresses immune disorders. Proc Natl Acad Sci. 2010;107(5):2159–2164. doi:10.1073/pnas.0904055107. 20080669 PMC2836639

[36] Sommer F, Bäckhed F. The gut microbiota-masters of host development and physiology. Nat Rev Microbiol. 2013;11(4):227–238. doi:10.1038/nrmicro2974. 23435359

[37] Rooks MG, Veiga P, Wardwell-Scott LH, Tickle T, Segata N, Michaud M, Gallini CA, Beal C, van Hylckama-vlieg JE, Ballal SA, et al. Gut microbiome composition and function in experimental colitis during active disease and treatment-induced remission. ISME J. 2014;8(7):1403–1417. doi:10.1038/ismej.2014.3. 24500617 PMC4069400

[38] Berry D, Reinisch W. Intestinal microbiota: A source of novel biomarkers in inflammatory bowel diseases? Best Pract Res Clin Gastroenterol. 2013;27(1):47–58. doi:10.1016/j.bpg.2013.03.005. 23768552

[39] Roopchand DE, Carmody RN, Kuhn P, Moskal K, Rojas-Silva P, Turnbaugh PJ, Raskin I. Dietary polyphenols promote growth of the gut bacterium *Akkermansia muciniphila* and attenuate high-fat diet-induced metabolic syndrome. Diabetes. 2015;64(8):2847–2858. doi:10.2337/db14-1916. 25845659 PMC4512228

[40] Ottman N, Reunanen J, Meijerink M, Pietila TE, Kainulainen V, Klievink J, Huuskonen L, Aalvink S, Skurnik M, Boeren S, et al. Pili-like proteins of *Akkermansia muciniphila* modulate host immune responses and gut barrier function. PLoS One. 2017;12(3):e0173004. doi:10.1371/journal.pone.0173004. 28249045 PMC5332112

[41] Louis P, Scott KP, Duncan SH, Flint HJ. Understanding the effects of diet on bacterial metabolism in the large intestine. J Appl Microbiol. 2007;102(5):1197–1208. doi:10.1111/j.1365-2672.2007.03322.x. 17448155

[42] Rather IA, Bajpai VK, Kumar S, Lim J, Paek WK, Park Y-H. Probiotics and atopic dermatitis: an overview. Front Microbiol. 2016;7:507. doi:10.3389/fmicb.2016.00507. 27148196 PMC4828648

[43] Lee SH, Yoon JM, Kim YH, Jeong DG, Park S, Kang DJ. Therapeutic effect of tyndallized *Lactobacillus rhamnosus* IDCC 3201 on atopic dermatitis mediated by down-regulation of immunoglobulin E in NC/Nga mice. Microbiol Immunol. 2016;60(7):468–476. doi:10.1111/1348-0421.12390. 27240551

[44] Grüber C, Wendt M, Sulser C, Lau S, Kulig M, Wahn U, Werfel T, Niggemann B. Randomized, placebo-controlled trial of *Lactobacillus rhamnosus* GG as treatment of atopic dermatitis in infancy. Allergy. 2007;62(11):1270–1276. doi:10.1111/j.1398-9995.2007.01543.x. 17919141

[45] Jang YJ, Kim WK, Han DH, Lee K, Ko G. *Lactobacillus fermentum* species ameliorate dextran sulfate sodium-induced colitis by regulating the immune response and altering gut microbiota. Gut Microbes. 2019;10(6):696–711. doi:10.1080/19490976.2019.1589281. 30939976 PMC6866707

[46] Kim W-K, Jang YJ, Seo B, Han DH, Park S, Ko G. Administration of *Lactobacillus paracasei* strains improves immunomodulation and changes the composition of gut microbiota leading to improvement of colitis in mice. J Funct Foods. 2019;52:565–575. doi:10.1016/j.jff.2018.11.035.

[47] Jimbo N, Kawada C, Nomura Y. Herb extracts and collagen hydrolysate improve skin damage resulting from ultraviolet-induced aging in hairless mice. Biosci Biotechnol Biochem. 2015;79(10):1624–1628. doi:10.1080/09168451.2015.1046362. 26011399

[48] Andoh T, Haza S, Saito A, Kuraishi Y. Involvement of leukotriene B4 in spontaneous itch-related behaviour in NC mice with atopic dermatitis-like skin lesions. Exp Dermatol. 2011;20(11):894–898. doi:10.1111/j.1600-0625.2011.01346.x. 21824199

[49] Kuraishi Y, Nagasawa T, Hayashi K, Satoh M. Scratching behavior induced by pruritogenic but not algesiogenic agents in mice. Eur J Pharmacol. 1995;275(3):229–233. doi:10.1016/0014-2999(94)00780-b. 7539379

[50] Livak KJ, Schmittgen TD. Analysis of relative gene expression data using real-time quantitative PCR and the 2^−ΔΔC^_ᵀ_ method. Methods. 2001;25(4):402–408. doi:10.1006/meth.2001.1262. 11846609

[51] Caporaso JG, Kuczynski J, Stombaugh J, Bittinger K, Bushman FD, Costello EK, Fierer N, Pẽa AG, Goodrich JK, Gordon JI, et al. QIIME allows analysis of high-throughput community sequencing data. Nat Methods. 2010;7(5):335–336. doi:10.1038/nmeth.f.303. 20383131 PMC3156573

[52] Kim W, Jang YJ, Han DH, Seo B, Park S. Administration of *Lactobacillus fermentum* KBL375 causes taxonomic and functional changes in gut microbiota leading to improvement of atopic dermatitis. Front Mol Biosci. 2019;6:1–12. doi:10.3389/fmolb.2019.00092. 31612141 PMC6777006

[53] Langille MGI, Zaneveld J, Caporaso JG, McDonald D, Knights D, Reyes JA, Clemente JC, Burkepile DE, Vega Thurber RL, Knight R, et al. Predictive functional profiling of microbial communities using 16S rRNA marker gene sequences. Nat Biotechnol. 2013;31:814–821. doi:10.1038/nbt.2676. 23975157 PMC3819121

[54] David LA, Maurice CF, Carmody RN, Gootenberg DB, Button JE, Wolfe BE, Ling AV, Devlin AS, Varma Y, Fischbach MA, et al. Diet rapidly and reproducibly alters the human gut microbiome. Nature. 2014;505(7484):559–563. doi:10.1038/nature12820. 24336217 PMC3957428

[55] Roucher VF, Desnots E, Naël C, Agnoux AM, Alexandre-Gouabau MC, Darmaun D, Boquien CY. Use of UPLC-ESI-MS/MS to quantitate free amino acid concentrations in micro-samples of mammalian milk. Springerplus. 2013;2:622. doi:10.1186/2193-1801-2-622. 24298434 PMC3841331

