# Supplementary material for: Lactobacillus paracasei KBL382 administration attenuates atopic dermatitis by modulating immune response and gut microbiota
Source: Gut Microbes. 2020 Oct 4;12(1):1819156. doi: 10.1080/19490976.2020.1819156 (PMC7553742; doi:10.1080/19490976.2020.1819156)
Supplement: Supplemental Material [file KGMI_A_1819156_SM6829.docx]

**
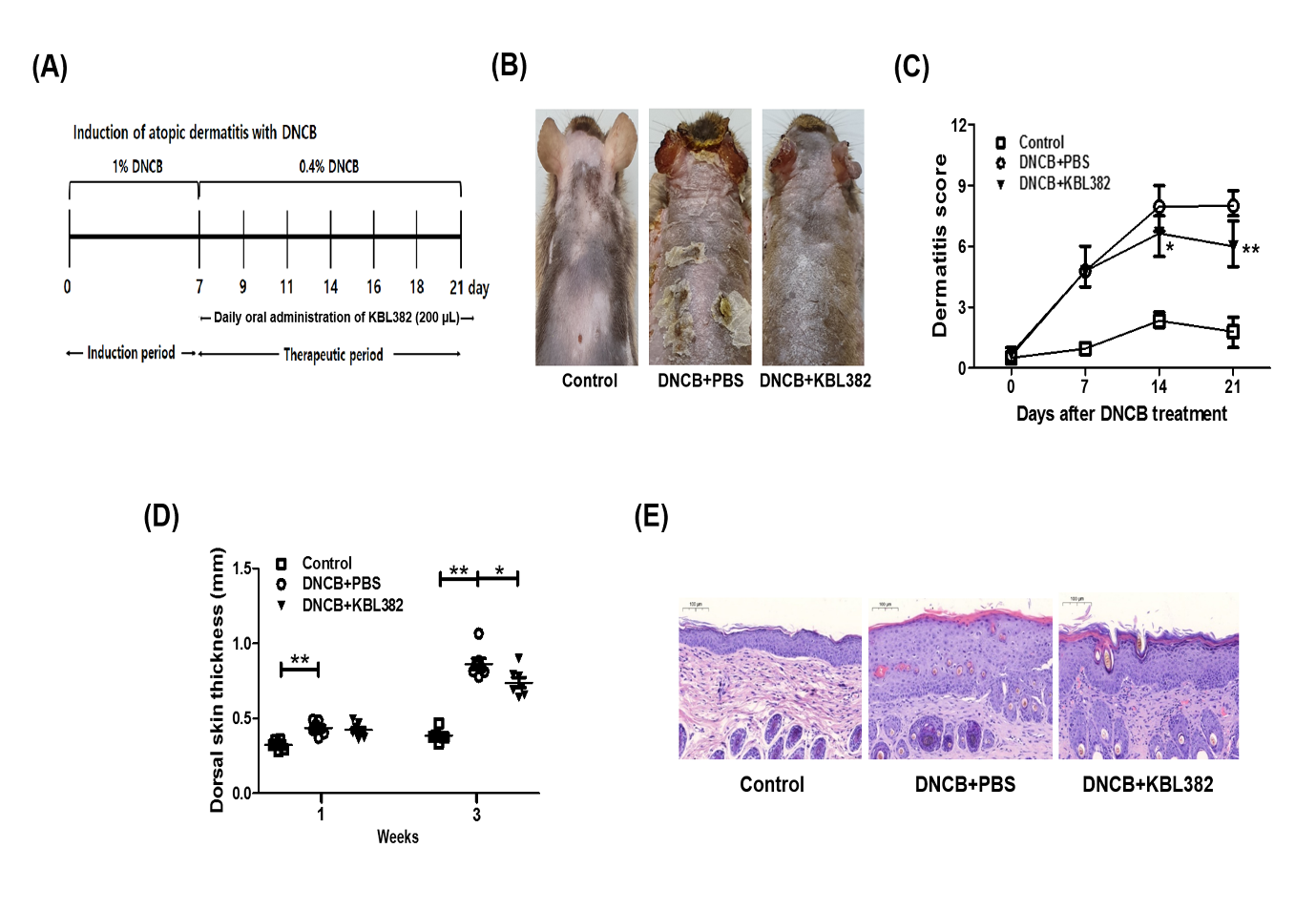
**

**Figure S1. Symptom changes with oral administration of *L*. *paracasei* KBL382 in mice with 2,4-dinitrochlorobenzene (DNCB)-induced atopic dermatitis (AD)-like symptoms.**

(A) Experimental design. The mice were treated with DNCB for 3 weeks. After 1 week from the initial DNCB application, mice were fed KBL382 for 2 weeks. Body weight and dermatitis scores were measured weekly. (B) Progress in DNCB-induced dermatitis in NC/Nga mice on d 21. Three groups of mice were treated with (1) PBS (Control), (2) DNCB+PBS, (3) DNCB+KBL382. (C) Dermatitis scores were evaluated weekly for 3 weeks. (D) Epidermis thickness was measured once a week for 3 weeks. (E) H&E staining of the dorsal skin lesions of mice on d 21. Statistical analysis was performed using the Mann-Whitney U test for comparison with DNCB+PBS mice (*N* = 6-7 mice per group). Error bars represent SEM. * *P* < .05; ** *P* < .01.

**Figure S2. Effects of KBL382 of IgE levels and cytokine expression in DNCB-induced AD skin lesions in NC/Nga mice.** The mice were sacrificed on d 21 and skin biopsy and serum collection were performed. (A) The ratio of spleen-to-body weight from sacrificed mice on d 21 was measured. (B) Serum level of IgE was measured using ELISA. Expression levels of cytokines genes related to (C) IL-4, (D) IL-5, (E) IL-13, (F) TNF-α, (G) IL-6, (H) IL-10 and (I) Foxp3 were evaluated by real-time PCR. Statistical analysis was performed using the Mann-Whitney U test for comparison with DNCB+PBS mice. Error bars represent SEM. * *P* < .05; ** *P* < .01; *** *P* < .001.


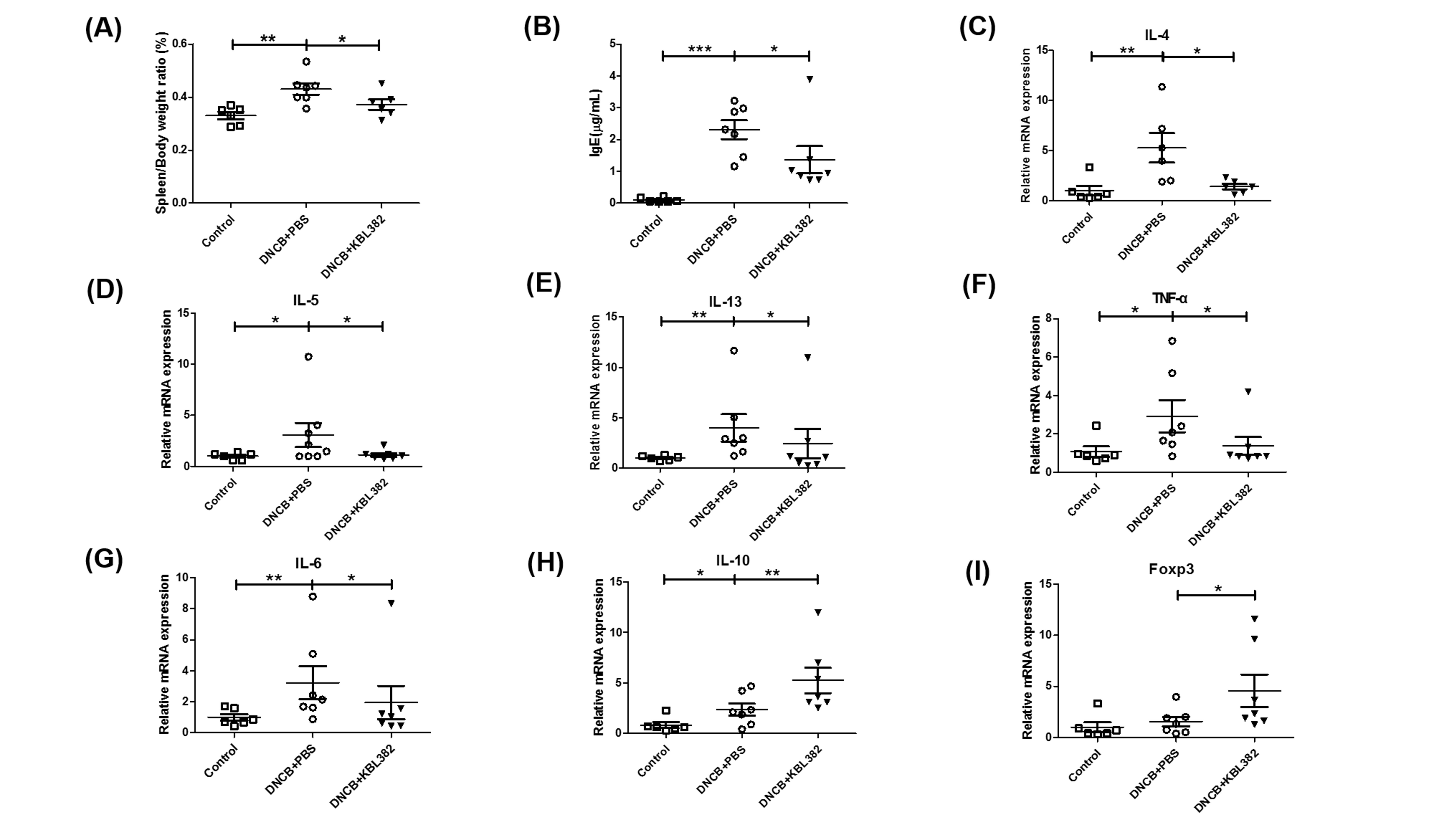


**Table S1.** Primers used in this study.

| Target | Sequence | Reference |
| --- | --- | --- |
| Foxp3 | Fw^a^: 5′- CCC ATC CCC AGG AGT CTT G -3′ | 1 |
|  | Rv^b^: 5′- CCA TGA CTA GGG GCA CTG TA -3′ |  |
| HPRT | Fw: 5′- TTA TGG ACA GGA CTG AAA GAC -3′ |  |
|  | Rv: 5′- GCT TTA ATG TAA TCC AGC AGG -3′ |  |
| IL-2 | Fw: 5′- CCT GAG CAG GAT GGA GAA TTA CA -3′ |  |
|  | Rv: 5′- TCC AGA ACA TGC CGC AGA G -3′ |  |
| IL-4 | Fw: 5′- ACA GGA GAA GGG ACG CCA T -3′ |  |
|  | Rv: 5′- GAA GCC CTA CAG ACG AGC TCA -3′ |  |
| IL-5 | Fw: 5′- TCC AAT GCA TAG CTG GTG ATT T -3′ |  |
|  | Rv: 5′- AGC ACA GTG GTG AAA GAG AC -3′ |  |
| IL-6 | Fw: 5’-CTG CAA GAG ACT TCC ATC CAG -3’ | 2 |
|  | Rv: 5’-AGT GGT ATA GAC AGG TCT GTT GG -3’ |  |
| IL-10 | Fw: 5′- TCA TTT CCG ATA AGG CTT GG -3′ | 1 |
|  | Rv: 5′- ATA ACT GCA CCC ACT TCC CA -3′ |  |
| IL-13 | Fw: 5′- GCA ACA TCA CAC AGG ACC AGA -3′ |  |
|  | Rv: 5′- GTC AGG GAA TCC AGG GCT AC -3′ |  |
| IL-31 | Fw: 5′-ATA CAG CTG CCG TGT TTC AG -3′ | 3 |
|  | Rv: 5′- AGC CAT CTT ATC ACC CAA GAA -3′ |  |
| MDC | Fw: 5'- CCA AGG TGC CTT TGA AGA CT -3' | 4 |
|  | Rv: 5'- TCC TCC AGC TGG TGG TTA CT -3' |  |
| TARC | Fw: 5'- CAG GAA GTT GGT GAG CTG GTA TA -3' | 5 |
|  | Rv: 5'- TTG TGT TCG CCT GTA GTG CAT A -3' |  |
| TGF-β | Fw: 5′- GAA GGC AGA GTT CAG GGT CTT -3′ | 1 |
|  | Rv: 5′- GGT TCC TGT CTT TGT GGT GAA -3′ |  |
| TNF-α | Fw: 5′- CAT CTT CTC AAA ATT CGA GTG ACA A -3′ |  |
|  | Rv: 5′- TGG GAG TAG ACA AGG TAC AAC CC -3′ |  |
| TSLP | Fw: 5'- CGA GCA AAT CGA GGA CTG TGA G -3' | 6 |
|  | Rv: 5'- GCA GTC GTC ATT GAG CGC TTC -3 |  |

^a^Fw represents sequences of a forward primer

^b^Rv represents sequences of a reverse primer

**References**

1. Kwon H-K, Lee C-G, So J-S, Chae C-S, Hwang J-S, Sahoo A, Nam JH, Rhee JH, Hwang K-C, Im S-H. Generation of regulatory dendritic cells and CD4+Foxp3+ T cells by probiotics administration suppresses immune disorders. Proc Natl Acad Sci. 2010;107(5):2159-64. doi:10.1073/pnas.0904055107.
2. Chang H-H, Miaw S-C, Tseng W, Sun Y-W, Liu C-C, Tsao H-W, Ho I-C. PTPN22 modulates macrophage polarization and susceptibility to dextran sulfate sodium-induced colitis. J Immunol. 191(5), 2134-43*.* doi:10.4049/jimmunol.1203363.
3. Takaoka A, Arai I, Sugimoto M, Honma Y, Futaki N, Nakamura A, Nakaike S. Involvement of IL-31 on scratching behavior in NC/Nga mice with atopic-like dermatitis. Exp Dermatol. 2006;15(3):161-7. doi:10.1111/j.1600-0625.2006.00405.x.
4. Kee J-Y, Jeon Y-D, Kim D-S, Han Y-H, Park J, Youn D-H, Kim S-J, Ahn KS, Um J-Y, Hong S-H. Korean Red Ginseng improves atopic dermatitis-like skin lesions by suppressing expression of proinflammatory cytokines and chemokines. J Ginseng Res. 2017;41:134-43. doi: 10.1016/j.jgr.2016.02.003.
5. Sung Y-Y, Yoon T, Jang S, Kim HK. *Forsythia suspensa* suppresses house dust mite extract-induced atopic dermatitis in NC/Nga mice. PLoS One. 2016;11(12):e0167687. doi:10.1371/journal.pone.0167687.
6. Zhao H, Li M, Wang L, Su Y, Fang H, Lin J, Mohabeer N, Li D. Angiotensin II induces TSLP via an AT1 receptor/NF-κB pathway, promoting Th17 differentiation. Cell Physiol Biochem. 2012;30(6):1383-97. doi:10.1159/000343327.
